# Enhancing phylloquinone levels using ultraviolet-A radiation in indoor farming

**DOI:** 10.1371/journal.pone.0319469

**Published:** 2025-04-10

**Authors:** Yuyao Kong, Krishna Nemali

**Affiliations:** 1 Department of Horticultural Sciences, Texas Agricultural and Mechanical University, College Station, Texas, United States of America; 2 Department of Horticulture and Landscape Architecture, Purdue University, Indiana, United States of America; Wageningen University, NETHERLANDS, KINGDOM OF THE

## Abstract

Phylloquinone (Phyllo) or vitamin K1 is mostly available in plant-based foods such as spinach and lettuce. Because Phyllo absorption in the human gut is low, foods with significantly high levels of Phyllo can aid in maintaining adequate vitamin K levels when consumed. We conducted two experiments, i.e., monochromatic and broadband, to understand the effects of light quality on enhancing Phyllo levels in lettuce. Both experiments used green romaine lettuce and a customized indoor growth system with light emitting diode (LED) lights. We measured fresh weight (FW), dry weight, leaf area, leaf number, and Phyllo levels in both experiments. Photosynthesis (A)- photon flux density (*PPFD*) response curves were measured in the second experiment. In the first experiment, plants were grown under six monochromatic light treatments viz., ultraviolet (UV_389_), blue (B_450_), green (G_521_), red (R_632_), hyper-red (R_662_), and far-red (FR_733_) during the entire growth period. Phyllo level was higher in UV_389_ and not different among other treatments. Vegetative growth parameters trended in the order of R_632_ > R_662_/G_521_ > B_450_ > UV_389_ > FR_733_. These results suggested that UV_389_ can increase Phyllo levels but its addition can have a negative effect on vegetative growth. In the second experiment, plants were grown under two treatments viz., UV_389_ substituted in the broadband light (40% of total light) during the stationary growth stage (UV_sub_) and control (broadband light without substitution). Results indicated that FW was lower by 24% but Phyllo level increased by approximately 175% in the UV_sub_ treatment compared to control. These results suggest that UV_389_ provided during the stationary growth phase can enhance Phyllo, however, further lowering the percentage of UV_389_ may be required to minimize the negative effect on vegetative growth. Analysis of A-*PPFD* curves indicated lower operating photosynthesis (A_OP_) and light use efficiency (LUE) in the UV_sub_ compared to control. Analysis indicated that UV-A light provided during the stationary growth stage contributed little to A_OP_. This suggests that increased Phyllo levels from UV-A exposure did not enhance A but likely provided photoprotection by channeling excess excitation energy through alternate pathways.

## Introduction

Light quality can affect the biosynthesis of secondary metabolites with nutritional value [1,2,3]. Crops with enhanced nutritional quality are preferred by consumers [4,5], which may aid in increasing their wholesale value. In recent years, an innovative method of farming called ‘indoor farming’ has become popular in many urban centers globally. This farming method involves growing mostly leafy greens [6] using controlled environmental conditions. LED-based lighting is used to grow plants in indoor farming due to their reduced thermal emissions and increased energy efficiency and flexibility with customization [7]. The LED lights provided to plants in indoor farming are usually customized to maximize vegetative growth. It may be possible to enhance the nutritional quality of crops grown in indoor farming by customizing LED light quality. Given this, many researchers focused on the effects of light quality on the biosynthesis of carotenoids, anthocyanins, flavonoids, and phenolics in crops [1,8–10] as they affect color and flavor. In this article, we describe the effects of light quality on the biosynthesis of Phyllo, which is relatively less studied.

Phylloquinone is the most common form of vitamin K consumed by humans [11]. In addition to its role in blood coagulation, Phyllo is important for bone health, cardiovascular health, metabolism, reproduction, and lowering cancer progression [12]. Among the commonly consumed foods, spinach, broccoli, and romaine lettuce are good sources of Phyllo [13]. Although vitamin K deficiency is rare, its depletion can lead to serious health-related issues. For example, vitamin K deficiency accelerated elastic fiber damage and thrombosis in COVID-19 patients [14]. Moreover, the absorption of Phyllo from plant-based foods (e.g., spinach) by the digestive system is generally low at 4–17% [15]. Therefore, maintenance of vitamin K levels in humans may require regular consumption of leafy greens or other plant-based foods. However, as there are no upper limits established for vitamin K consumption [16] the normal levels may be maintained by consuming plant-based foods with enhanced levels of Phyllo, especially in those who occasionally consume leafy greens.

Phylloquinone is primarily found in the photosynthetic tissue of plants and concentrated in chloroplasts [17]. It is a cofactor of photosynthetic phosphorylation [18] and serves as an electron carrier for PSI [19]. A recent study reported that phylloquinone is the principal site for the Mehler reaction within PSI [20], which uses molecular oxygen as a sink for excess electrons generated in the photosynthetic machinery when plants are exposed to high-energy radiation such as UV [21]. Given this, Phyllo may also aid in photoprotection against high-energy radiation such as UV in addition to its role in photosynthetic phosphorylation.

High-energy UV radiation may increase Phyllo levels but is not used as part of LED lighting in indoor farming due to negative effects on vegetative growth. Ultra-violet light can be classified into three categories, including UV-A (320–400 nm), UV-B (280–320 nm), and UV-C (200–280 nm), which represent 6.3%, 1.5%, and 0.5% of the total solar energy received, respectively [22,23]. The UV-A wavelengths (380–400 nm) are absorbed by plant pigments such as phototropins and cryptochromes [24,25]. Due to relatively higher energy levels, exposing plants to UV-A was shown to increase phytochemical levels [26,27] likely for photoprotection. However, several studies reported that UV-A radiation reduced quantum yield and photosynthesis [28], and leaf expansion and biomass [29,30]. The effects of long-term supplementation of UV radiation on plant biomass depended on wavelength, intensity, and species [26,27,31,32].

The negative effect of UV-A on vegetative grwth may be smaller if the exposure is for a shorter period. Vegetative growth generally has three stages including the lag phase, logarithmic phase, and stationary or mature phase [33]. In general, maximum vegetative growth is seen during the logarithmic phase with slower growth rates observed during the lag and stationary phases [34]. The addition of UV lighting during the lag phase may stunt the overall growth and negatively affect the expected rapid growth during the log phase [35]. Therefore, it is more logical to provide UV radiation for a short period during the stationary phase. Moreover, the effect of spectral quality on the biosynthesis of phytochemicals was found to be higher during the stationary phase [36,37].

However, the following questions need to be addressed before recommending the application of UV lighting in indoor farming: (i) is UV-A the only waveband that can enhance Phyllo levels in plants? (ii) can a short-period exposure to UV-A during stationary growth enhance Phyllo levels? (iii) what is the role of increased levels of Phyllo, increased photoprotection or photophosphorylation? Keeping these questions in mind, the following objectives were set for study: (i) quantify the effect of monochromatic wavebands of UV_389_, B_450_, G_521_, R_632_, R_662_, and FR_733_ (subscripts refer to peak wavelength of each waveband) on Phyllo biosynthesis and vegetative growth to understand the independent effects of light quality on Phyllo biosynthesis and aid in providing recommendations on customized light spectrum for indoor farming, (ii) study the effect of short-term (7 days) exposure of UV-A during the stationary growth period on Phyllo biosynthesis and vegetative growth, and (iii) understand the effects of enhanced Phyllo levels on photoprotection and photophosphorylation.

## Materials and methods

We chose green romaine lettuce (*Lactuca sativa* cv. Amadeus) in our research as it is a popular choice for salads and has higher levels of Phyllo than the other leafy greens [13]. Two separate experiments were conducted in our study viz., monochromatic and broadband experiments. The monochromatic experiment compared the long-term effects of exposing lettuce plants to different monochromatic waveband treatments whereas the broadband experiment studied the short-term effects of substituting UV-A radiation in the broadband light during the stationary growth period. In addition, data from experiments were used to understand the interrelations between Phyllo and photosynthesis/ vegetative growth.

### Plant material

In both experiments, seeds of green romaine lettuce plants (*Lactuca sativa*) cultivar Amadeus were germinated in plug flats (72-cell; 3.5 cm ×  3.5 cm ×  5.9 cm, 30.2 mL per cell, Landmark Plastic, Akron, Ohio, USA) filled with a soilless substrate (80% peat, 15% perlite, and 5% vermiculite, BM-2, Berger, Saint Modeste, Canada). Seedlings were transplanted into square pots (10.6 cm ×  10.6 cm ×  8.4 cm, 943 mL, Kord Products Ltd, Brampton, ON, Canada) filled with the same soilless substrate and grown in an indoor growth room as described below.

### Production system

The experiments used a custom-built indoor production system in a walk-in growth room. The production system was built using chrome-wire shelves [1.22 m (length) ×  0.61 m (width) ×  1.83 m (height), Amazon, USA), hydroponic trays (1.22 m ×  0.61 m ×  0.1 m, Active Aqua Co., Hydrofarm.com, USA), and LED fixtures. Each shelf had four levels spaced 0.35 m apart. Each level was divided into two growth units (0.61 m ×  0.61 m) using a white reflecting barrier and each unit housed four lettuce plants (15 cm spacing) in the center. The LED fixtures (Total Grow, MI, USA; Happy Leaf LLC, USA) were installed at the top of each growth unit on the shelves. The distance between the top of the plants and the LED lights was 20 cm.

### Growing conditions

The temperature in the growth room was controlled by an air conditioner (OWC1811, OceanAire, IL, USA). The average temperature and relative humidity in the monochromatic and broadband experiments were 27.5 ±  1.62 °C/ 64 ±  7.4% and 24.3 ±  1.09/ 72 ±  3.7% respectively. In both experiments, an average light intensity of approximately 200 µmol ∙ m^-2^ ∙ s^-1^ was provided but the spectral composition varied by treatment (see Table 1 and treatments section below). The light intensity was adjusted using an LED light strip dimmer (DC 12V-24V 30A PWM Dimming Controller, Amazon, USA) connected to the light fixture. The photoperiod for both experiments was set to 24 hours. Plants were watered with a nutrient solution (20-10-20, Peters Professional, Summerville, SC, USA), which was recycled between growing trays and reservoirs for 30 min each day, during which the substrate absorbed the nutrient solution due to capillary action (sub-irrigation). In both experiments, the electrical conductivity (EC) and pH of the nutrient solution were maintained at 1.62 ±  0.032 dS·m^-1^ and 6.5 to 6.8, respectively. Plants were grown for 28 days in both experiments.

**Table 1:** Average light intensity and spectral quality in the monochromatic and broadband substitution experiments. UV-A = ultraviolet A (340-399 nm), B = blue (400-499 nm), G = green (500-599nm), R = red (600-699 nm), and FR = far red (700-750 nm).

Experiment	Treatment	Light Intensity	Spectral Quality				
			UV-A	B	G	R	FR
		(µmol·m-2·s-1)			(%)		
Monochromatic							
	UV_389_	195.7 ± 11.6	100	0	0	0	0
	B_450_	188.5 ± 4.96	0	100	0	0	0
	G_521_	196.8 ± 7.56	0	0	100	0	0
	R_632_	193.4 ± 6.08	0	0	0	100	0
	R_662_	191.8 ± 8.34	0	0	0	100	0
	FR_733_	191.1 ± 9.96	0	0	0	0	100
Broadband	UV_sub_	204.0 ± 1.79	40	4	13	43	0
	Control	202.8 ± 2.36	0	7	21	72	0

### Treatments

**Fig 1:**
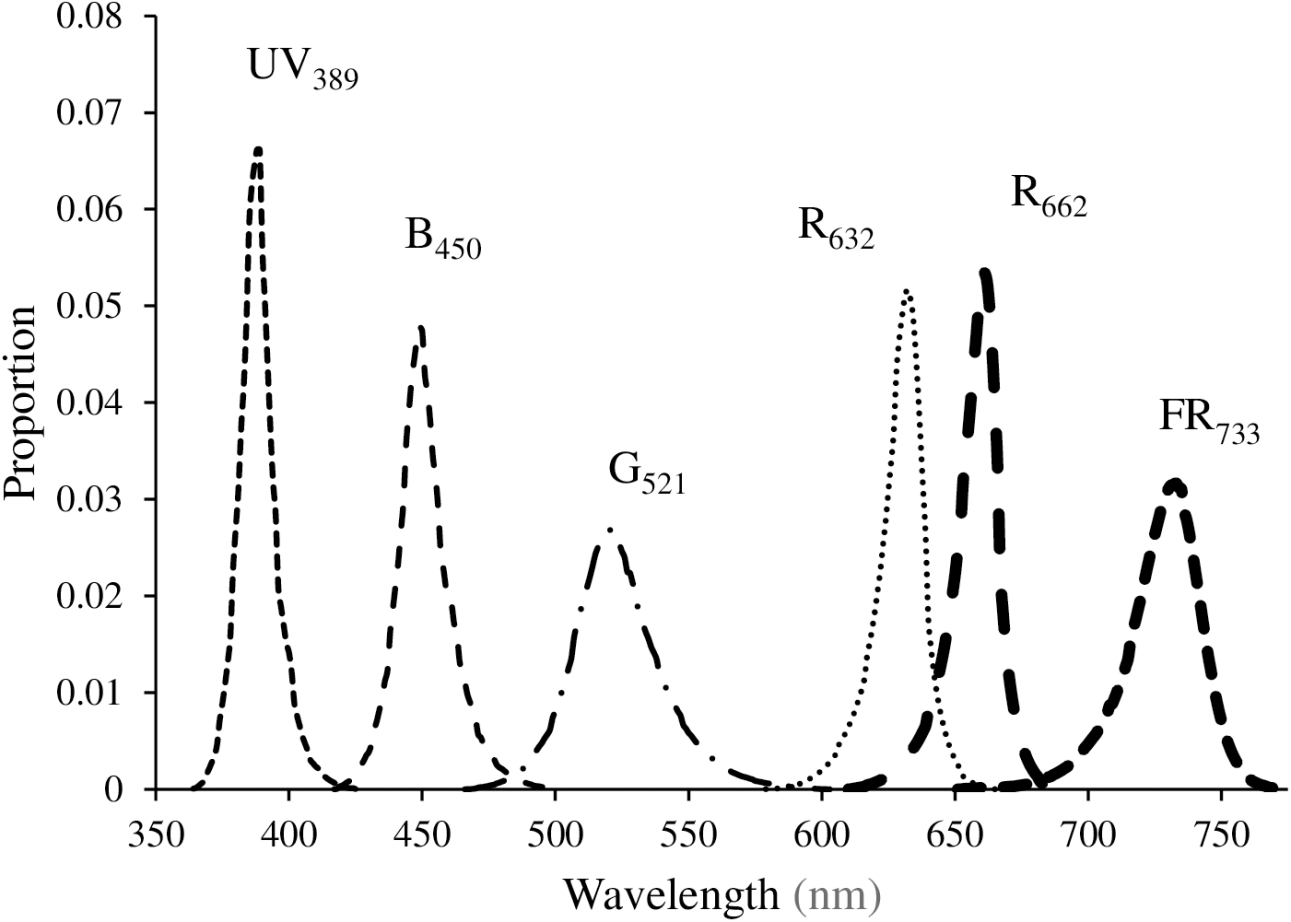
Spectral quality of different waveband treatments in the monochromatic experiment.

**Fig 2:**
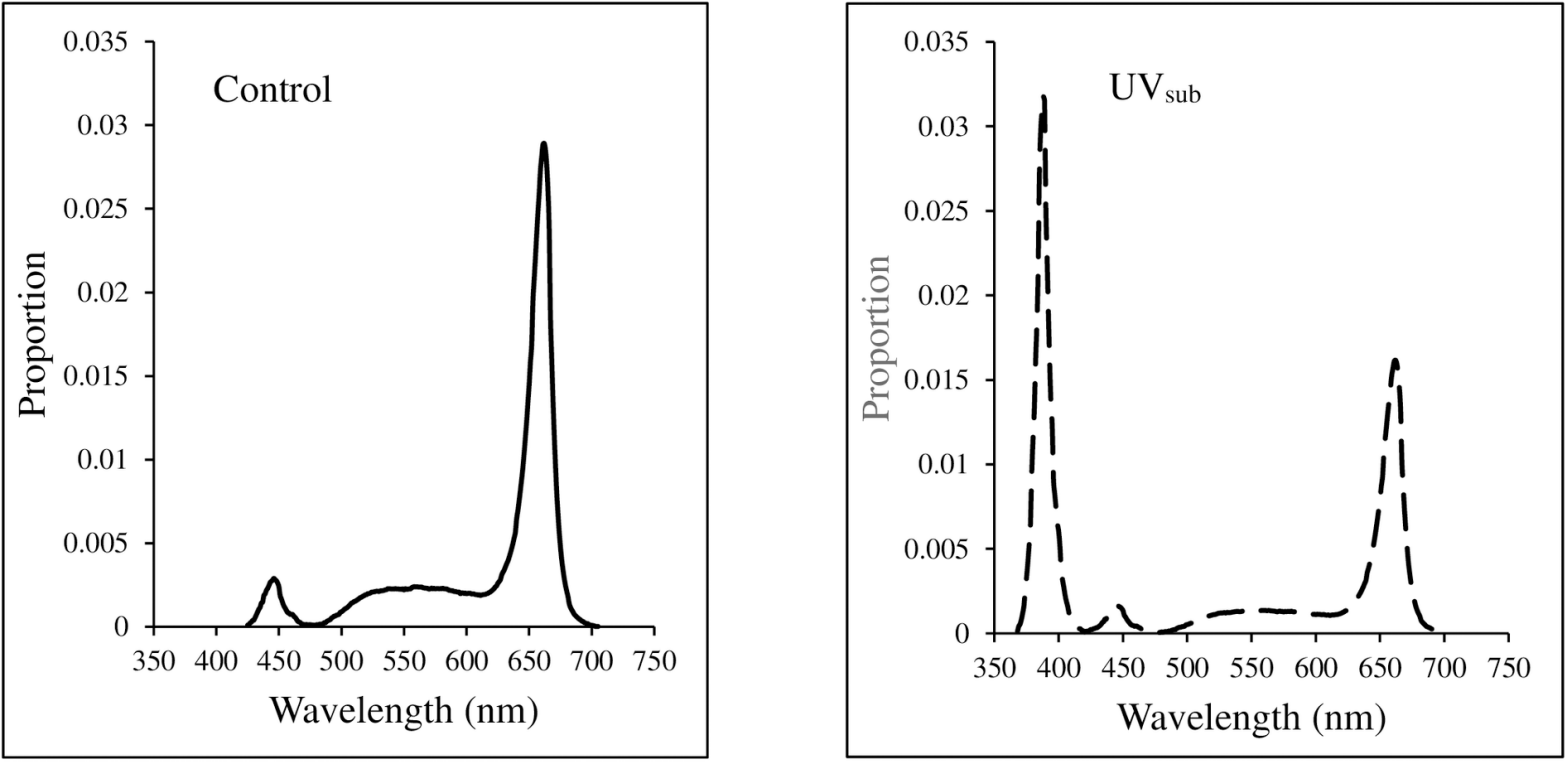
Spectral quality of broadband ‘control’ and UV-A substituted broadband light (UV_sub_) treatments. Plants in broadband treatment received the same spectral quality during the growth whereas plants in the UV_sub_ treatment received broadband light for 21 days and UV-A substituted broadband light during the 7 days before harvest.

In the monochromatic experiment, plants were exposed to long-term light treatments containing solely UV_389_ (control), B_450_, G_521_, R_632_, R_662_, and FR_733_ for the entire growth period (Table 1, Fig 1). The wavebands ranged from 365–420 (UV_389_), 420–500 (B_450_), 475–580 (G_521_), 590–650 (R_632_), 615–690 (R_662_), and 670–770 (FR_733_) nm with a full width at half maximum (FWHM) between 25–50 nm. The FWHM was narrower for UV_389_, intermediate for B_450_, R_632_, and R_662_, and wider for G_521_ and FR_733_ treatments (Fig 1). In the broadband experiment, plants were exposed to two light treatments viz., control (continuous broadband light during growth) and UV_sub_ (broadband light substituted with 40% of UV_389_ for a short-term (7 days) during the stationary growth phase) (Table 1, Fig 2). A high (40 as opposed to 10–15%) percentage of UV substitution was chosen to ensure that the effects of UV are observed during the short exposure period of seven days. The spectral quality of UV_sub_ treatment was changed during the seven days before harvest by lowering the intensity of broadband light to approximately 120 µmol ∙ m^-2^ ∙ s^-1^ and adding 80 µmol ∙ m^-2^ ∙ s^-1^ of UV_389_ light from separate UV LED bars. The UV_389_ substitution resulted in a similar (40%) reduction in the intensities of blue, green, and red wavebands.

### Measurements

Air temperature and relative humidity in the growth room were measured continuously using a thermistor (ST-100, Apogee Instruments, Logan, UT, USA) and a relative humidity probe (EE08-SS, Apogee Instruments, Logan UT, USA) connected to a datalogger (CR1000, Campbell Scientific, Logan, UT, USA). The average light intensity was measured at five different locations in each experimental unit using a quantum sensor (SQ-620-SS, Apogee Instruments, Logan, UT, USA). Light quality was measured using a spectroradiometer at the center of each experimental unit (SS-110, Apogee Instruments, Logan, UT, USA). The EC and pH of the nutrient solution were measured by a portable pH/EC/TDS meter (Hanna Instruments, Chico, CA, USA).

To assess plant growth, lettuce shoot FW (g·plant^-1^), shoot dry weight (DW, g·plant^-1^), total leaf area (LA, cm^2^·plant^-1^), and leaf number (LN) were measured in both experiments. Two representative plants from each experimental unit were harvested at the base to measure the FW. Leaves belonging to a plant were separated and counted to get the leaf number (LN). Leaf number was counted to assess whether shade-avoidance responses are present in plants exposed to monochromatic light treatments. Then the separated leaves were run through the rollers of a leaf area meter (LI-3100C, Li-Cor Biosciences, Lincoln, NE, USA) to measure the total LA. Finally, the separated leaves belonging to a plant were collected in a paper bag and placed in a forced-air oven maintained at 70 °C until dry to measure DW.

Photosynthesis (A) response to increasing *PPFD* levels was measured in the broadband experiment on the day before harvest. The leaf photosynthesis was measured on a fully expanded leaf at the top of the canopy using a portable open-flow leaf photosynthesis system (LI-6400XT, Li-Cor Biosciences, Lincoln, NE, USA). A clear-top leaf chamber was used to measure the leaf photosynthesis rate between 10 am and 2 pm on a given day. This allowed the photosynthesis measurements under the same light quality incident on the plants in a given treatment. The CO_2_ concentration, RH, and air temperature inside the leaf chamber were set to 400 µmol·mol^ − 1^, 60%, and 25°C, respectively, during the measurement. The readings were recorded when the gas exchange rate was stable. Plants were exposed to eight light intensities ranging from 0 to 500 µmol·m^-2^·s^-1^. The maximum light intensity was set to 500 µmol·m^-2^·s^-1^ as ambient light was used to generate different light levels and higher intensities were not possible from the LED light fixtures. The measurements started at the highest light intensity level. After the measurement, the light intensity was decreased to the next lower level using a dimmer/ controller. This process continued until the measurements were completed for all light intensity levels (500, 400, 300, 200, 100, 50, 25, 0 µmol·m^-2^·s^-1^). The following quadratic equation was fitted to A-*PPFD* curves data:


$$A=c+\left(b\times PPFD\right)-\left(a\times PPFD^{2}\right)$$

Where a (intercept) is the dark respiration rate (R_d_), and b and c are slope constants. The A_OP_ at a *PPFD* of 200 µmol·m^-2^·s^-1^ (i.e., intensity to which plants were exposed during growth) was calculated by substituting a, b, c, and *PPFD* values in the above equation. Light use efficiency (defined as moles of CO_2_ fixed per mole of *PPFD*) was calculated as the first-derivative of the fitted quadratic equation at a *PPFD* of 200 µmol·m^-2^·s^-1^ as follows:


$$LUE=\frac{d}{dx}\left(A\right)=b-2\times c\times PPFD$$

Light compensation point (LCP, defined as the minimum *PPFD* for positive photosynthesis) was calculated by solving the fitted quadratic equation when A = 0 as follows:


$$LCP=\frac{-b+\sqrt{b^{2}-4ac})}{2a}$$

Phylloquinone (Phyllo) levels were measured on a fresh weight basis. For Phyllo measurements, the leaf samples collected on the harvest day were ground in the dark into a fine powder with liquid nitrogen. Then 1 g of the ground tissue was weighted and moved into a 15 ml falcon tube (Fisher Scientific, USA) wrapped with aluminum foil to avoid photodegradation. The samples were sent to a laboratory (Bindley Bioscience Center, Purdue University, USA) for Phyllo analysis using liquid chromatography/ mass spectrometry (Agilent 6460 Triple Quadrupole LC/MS system, Agilent Technologies, CA, USA). Samples were homogenized and Phyllo was initially extracted using an organic solvent mixture followed by solid phase extraction for further purification. For separation, a reverse-phase HPLC method was used. The Phyllo was detected and quantified using the mass spectrometer. A standard curve was constructed using known concentrations of phylloquinone to quantify the levels in the sample accurately.

### Experimental design and statistical analyses

In both experiments, a randomized complete block design with four replications was used. An experimental unit comprised a set of four plants belonging to a light treatment in a replication. Data were analyzed using a general linear model (GLM) procedure of statistical analysis software (SAS version 9.4, Cary, NC). A separate quadratic equation was fitted to the A-*PPFD* response data from each replication using the REG procedure of SAS. The parameters including a, b, c, A_OP_, and LUE were calculated for each replication using the fitted equation and analyzed using ANOVA (GLM) procedure of SAS. The normality of data was tested using the univariate procedure (Shapiro-Wilk test and Q-Q plot) and the homogeneity of variances was assessed by plotting residuals versus predicted values. The least-square means were separated using Tukey’s honestly significant difference (HSD) procedure in both experiments. A pre-determined alpha value of 5% (*P*-value ≤  0.05) was considered statistically significant for all analyses.

## Results

### Monochromatic experiment

**Fig 3:**
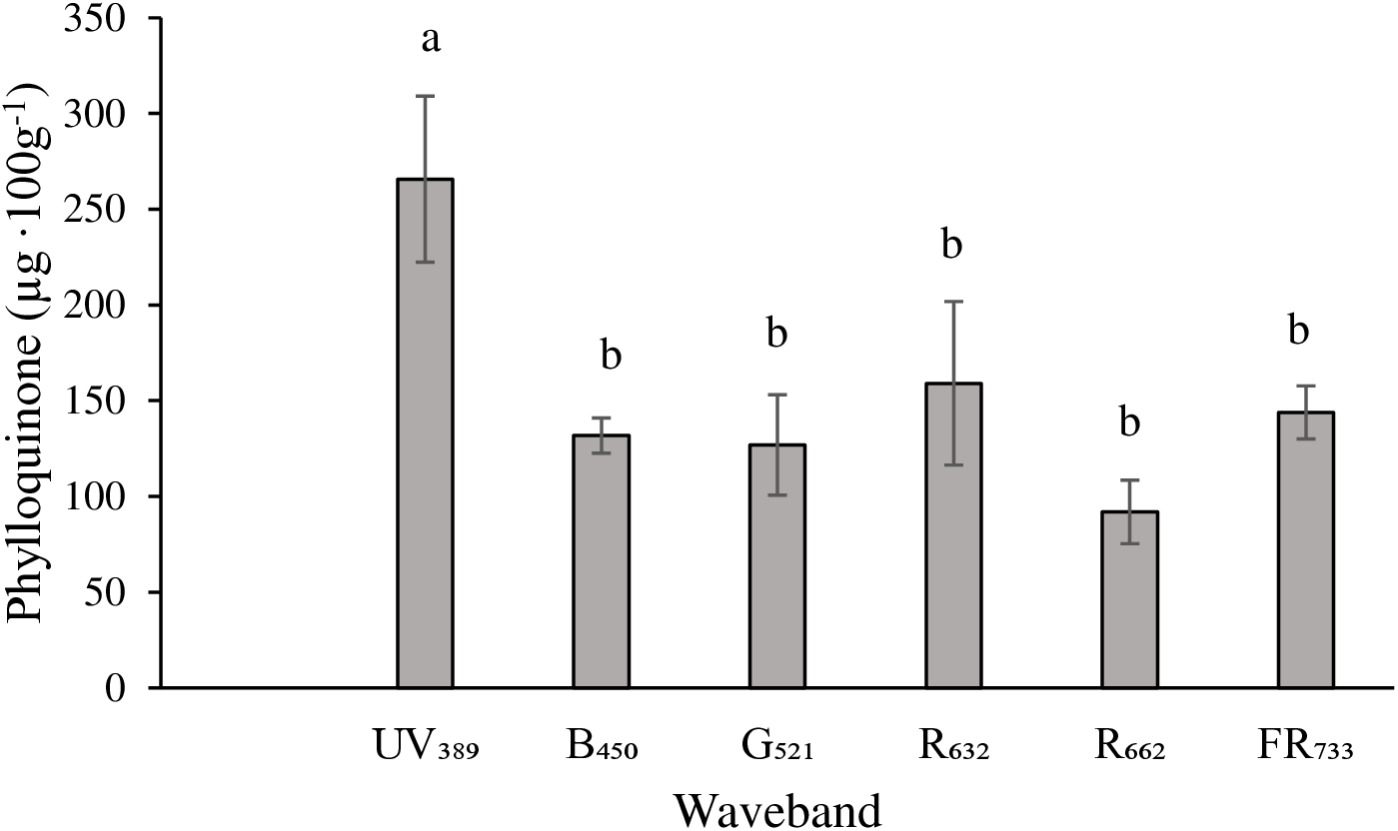
Effect of monochromatic waveband treatments on the levels of phylloquinone in green romaine lettuce cultivar Amadeus. Plants were grown in a custom indoor production system and exposed to six monochromatic light treatments. Data represents an average of four replications. Tukey HSD procedure was used to separate the least square means. Means (with standard errors) followed by different letters are statistically different at ****P**** ≤  0.05.

Statistically significant differences (*P* =  0.0095) in Phyllo levels of romaine lettuce cultivar Amadeus were observed among the treatments (Fig 3). Phylloquinone levels were significantly higher (265.7 µg ∙ 100 g^-1^) in UV_389_ treatment than in the other treatments. There were no statistical differences in Phyllo levels among the other treatments and their mean values ranged between 91.9 to 159.1 µg ∙ 100 g^-1^.

**Fig 4:**
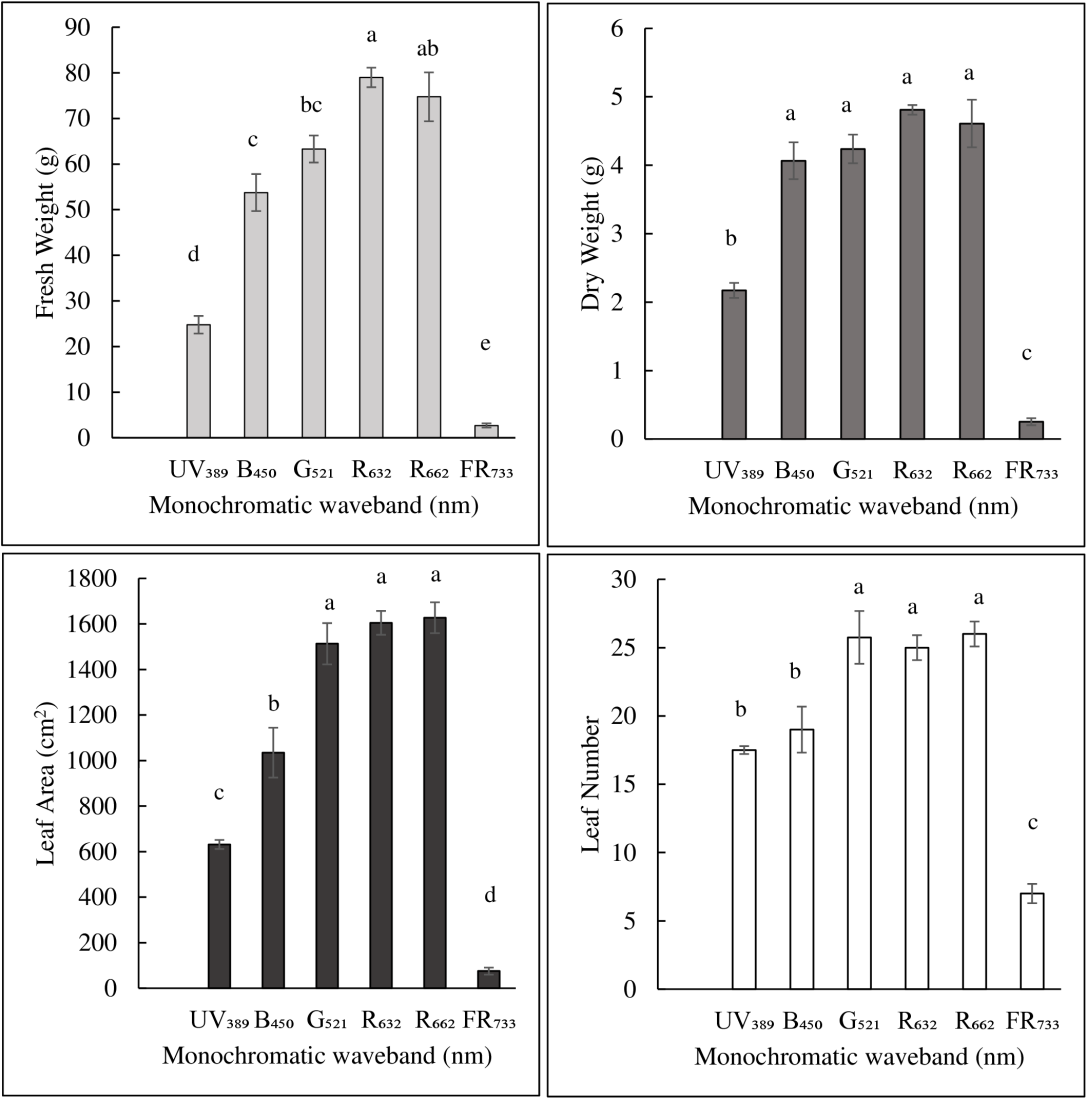
Effect of different monochromatic waveband treatments on vegetative growth characteristics in romaine lettuce. Plants were grown in a custom indoor production system and exposed to six monochromatic light treatments. Data represents an average of four replications. Tukey HSD procedure was used to separate the least square means. Means (with standard errors) followed by different letters are statistically different at ****P**** ≤  0.05.

The vegetative growth characteristics of romaine lettuce cultivar Amadeus including FW, DW, and LA were significantly different among treatments (Fig 4). While these characteristics were not generally different among R_632_, R_662_, and G_521_ treatments, they significantly decreased in other treatments in the order of B_450_>  UV_389_>  FR_733_. Lettuce FW was significantly affected by the monochromatic light treatment (*P* <  0.0001). It was the highest and not different between R_632_ and R_662_. The FW was higher in R_632_ than G_521_, B_450_, UV_389_, and FR_733_ treatments. There were no differences in FW between R_662_ and G_521_ and between G_521_ and B_450_ treatments. The FW in B_450_ was greater than UV_389_ which was higher than FR_733_ treatment. Lettuce DW was significantly affected by the monochromatic light treatment (*P* <  0.0001). The DW of lettuce was highest and not statistically different among B_450_, G_521_, R_632_, and R_662_ treatments. The DW of UV_389_ was lower than the above four treatments but higher than that of the FR_733_ treatment. Lettuce LA was significantly affected by the monochromatic light treatment (*P* <  0.0001). The LA was highest and not different among G_521_, R_632_, and R_662_ treatments. The LA of B_450_ was greater than UV_389_ which was higher than FR_733_ treatment. Lettuce LN was significantly affected by the monochromatic light treatment (*P* <  0.0001). Leaf number differences were similar to that of LA except that there were no differences between B_450_ and UV_389_ treatments.

**Fig 5:**
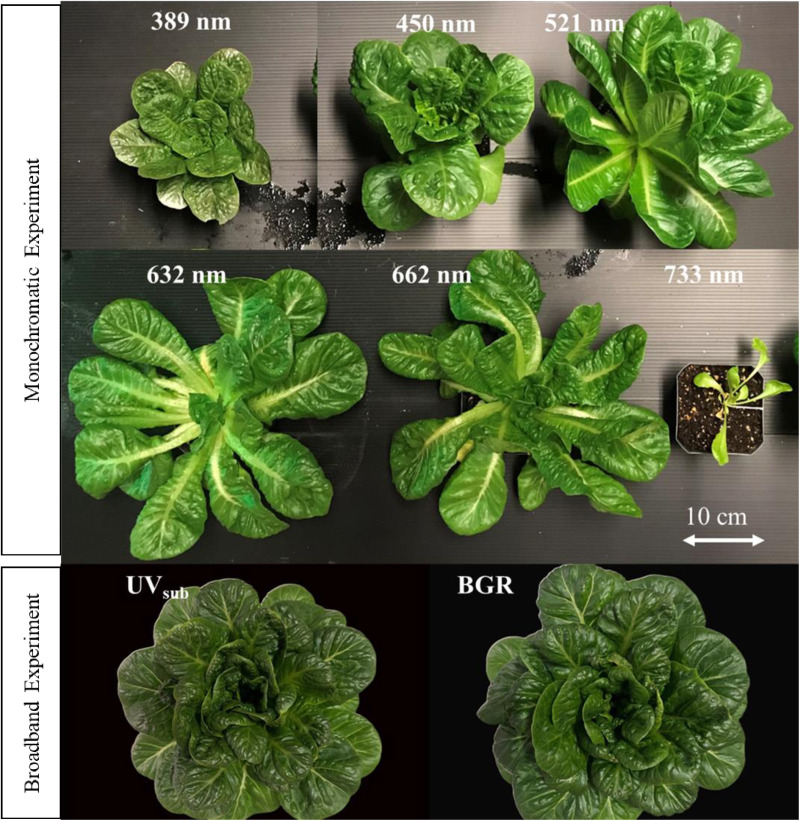
Representative romaine lettuce cultivar Amadeus plants from monochromatic (top) and broadband experiments (bottom) at the harvest stage. In the monochromatic experiment, plants were exposed to six different light treatments including ultra violet (389 nm), blue (450 nm), green (521 nm), red (632 and 662 nm) and far red (733 nm) wavebands. In the broad band experiment, plants were exposed to UV_sub_ (short-term substitution of 40% UV-A in the broadband light) and control (BGR; no substitution) treatments during the stationary growth stage.

Qualitative differences in plant morphology, leaf elongation, and chlorosis of romaine lettuce cultivar Amadeus were observed among the treatments (Fig 5**, top)**. Plants in the UV_389_ and B_450_ appeared compact with smaller leaves whereas elongation of petioles and leaves was evident in the monochromatic wavebands of G_521_, R_632_, and R_662_ treatments. The plants were small and biologically insignificant in the FR_733_ treatment. Petiole and leaf elongation was observed in the FR_733_ treatment. Leaves were chlorotic in both UV_389_ and FR_733_ treatments.

### Broadband experiment

Phylloquinone levels in romaine lettuce cultivar Amadeus were significantly different between the two treatments (Table 2). Phylloquinone levels were significantly higher in the UV_sub_ than control treatment. An increase of approximately 174% in Phyllo was observed in UV_sub_ than control (598.1 vs 217.5 µg ∙ 100 g^-1^) treatment. Moreover, the short-term substitution of broadband light with 40% of UV_389_ light during the stationary growth phase significantly decreased FW but not DW (Table 2). Lettuce grown in the control treatment had higher FW than those UV_sub_ treatment. The FW was higher by approximately 24% in the control than in the UV_sub_ treatment. There were no significant differences in DW between the UV_sub_ and control treatments.

**Table 2:** Effect of UV_389_ substitution of broadband light during stationary growth stage on fresh weight (FW), dry weight (DW), and phylloquinone level (Phyllo) in green romaine lettuce cultivar Amadeus exposed to UV_sub_ (short-term substitution of 40% UV-A in the broadband light) and control (no substitution) treatments during the stationary growth stage. Means (standard errors) followed by different letters are statistically different at *P* ≤  0.05.

Treatment	FW	DW	Phyllo
	g·plant^-1^	g·plant^-1^	µg·100g^-1^
UV_sub_	66.6 (1.89) b	4.9 (0.14) a	598.1 (58.05) a
Control	87.4 (3.60) a	5.6 (0.31) a	217.5 (18.45) b
*P*-value	0.0016	0.0749	0.0013

There were no significant differences in plant morphology and quality of romaine lettuce Amadeus between the two treatments in the broadband experiment (Fig 5**, bottom**). Green romaine lettuce plants in the UV_sub_ treatment did not show any chlorosis symptoms. Moreover, they were similar in plant morphology compared to the plants in the control treatment. However, the overall size of plants in UV_389_ appeared slightly smaller than the control plants.

**Fig 6:**
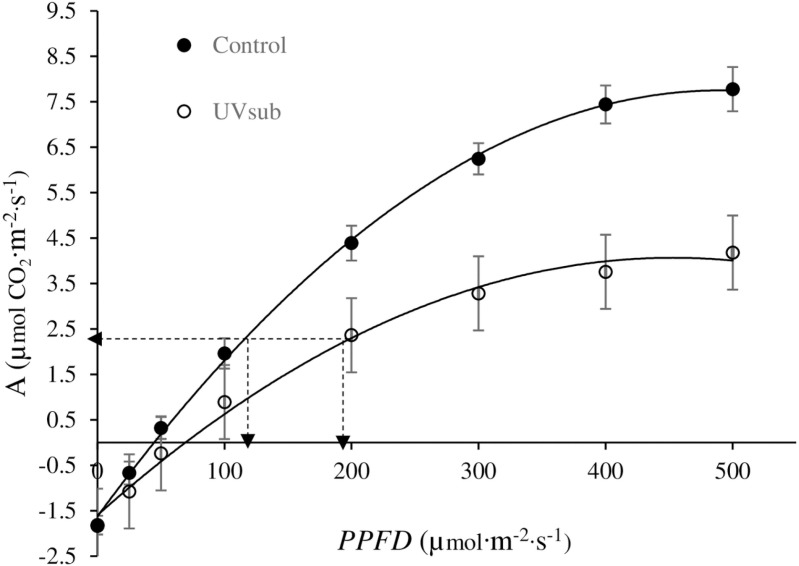
Photosynthesis (A) - response to increasing photosynthetic photon flux density (*PPFD*) in UV_sub_ and control treatments in the broadband experiment on the day before harvest. Data represents an average of four replications. Error bars represent the standard error of the mean. The fitted equations are A_(control)_ = −1.63 +  0.038 ∙ *PPFD* −0.00004 ∙ *PPFD*^2^ (*r*^2^ = 0.96) and A_(UVsub)_ = −1.60 +  0.025 ∙ *PPFD* -0.00003 ∙ *PPFD*^2^ (*r*^2^ = 0.90). The dashed lines indicate A at 120 and 200 µmol·m^-2^·s^-1^ of *PPFD* in the control and UV_sub_ treatments respectively.

The A*-PPFD* curves of romaine lettuce cultivar Amadeus were significantly different between the treatments in the broadband experiment (Fig 6). In general, the curves indicated a close relationship between the A and *PPFD* in both UV_sub_ and control treatments. The net photosynthetic rate increased with the increases in *PPFD* from 0 to 300 µmol·m^-2^·s^-1^ and further increases in *PPFD* from 300 to 500 µmol·m^-2^·s^-1^ resulted in smaller A increases in both treatments. However, the A was consistently higher in the control than that in UV_sub_ treatment starting from a *PPFD* level of approximately 50 µmol·m^-2^·s^-1^.

Among the estimated photosynthetic parameters of romaine lettuce Amadeus, both R_d_ (-1.63 vs -1.60 µmol·m^-2^·s^-1^) and LCP (45.5 vs 72.7 µmol·m^-2^·s^-1^) were not statistically different between the control and UV_sub_ treatments in (Table 3). However, both A_OP_ (4.5 vs 2.3 µmol·m^-2^·s^-1^) and LUE (0.023 vs 0.014 mol·mol^-1^) were significantly higher in the control than UV_sub_ treatment.

**Table 3:** Parameters estimated from the photosynthetic light response curves in the broadband substitution experiment for UV_sub_ (short-term substitution of 40% UV-A in the broadband light) and control (no substitution) treatments during the stationary growth stage, including operating photosynthetic rate (A_OP_), dark respiration rate (R_d_), light compensation point (LCP), and light use efficiency (LUE). Means (standard errors) followed by different letters are statistically different at *P* ≤  0.05.

Treatment	A_OP_	R_d_	LCP	LUE
	(µmol·m^-2^·s^-1^)	(µmol·m^-2^·s^-1^)	(µmol·m^-2^·s^-1^)	(mol·mol^-^^1^)
UV_sub_	2.3 (0.32) b	-1.60 (0.202) a	72.7 (13.22) a	0.014 (0.0011) b
Control	4.5 (0.35) a	-1.63 (0.123) a	45.5 (5.07) a	0.023 (0.0008) a
*P*-value	0.0014	0.8211	0.0736	0.0012

## Discussion

### Effects of monochromatic wavebands on Phyllo levels and vegetative growth

In our study, higher (approximately 1.7 to 2.8 times) levels of Phyllo were seen in the UV_389_ treatment (Fig 3) while the levels were lower and not different in other monochromatic wavebands. Therefore, the UV_389_ waveband appears to be the best choice among the tested wavebands for increasing Phyllo levels. Exposure to high intensity of UV-A radiation increases oxidative stress in plants [26]. The increase in Phyllo levels in the UV_389_ treatment in our study may be a response to oxidative stress experienced by plants. Interestingly, a recent study reported that Phyllo is the principal site for the Mehler reaction, an alternate path for high-energy electrons to avoid damage to the photosynthetic machinery [21], within PSI under high light conditions [20]. On the other hand, low-energy radiation such as FR light generally decreases the levels of phytochemicals and promotes elongation/ vegetative growth in plants [10,38]. However, the levels of Phyllo didi not decrease in FR_733_ compared to other monochromatic treatments (except UV_389_). Far-red light is also known to specifically activate PSI [39,40] and Phyllo is an integral component of PSI [19]. It is possible that Phyllo levels were maintained in the FR_733_ treatment to sustain the increased activity of PSI from FR exposure.

It is well-known that both light absorption and quantum yield affect vegetative growth. Light absorption and biomass production are positively correlated with leaf area [24,41,42]. This is likely the reason for the similar trends in FW and LA among different monochromatic treatments in our study. In his classic work, [28] showed that the relative quantum yield was highest in the monochromatic red waveband compared to the green and blue wavebands, and was significantly lower in the UV and far-red wavebands. Interestingly, the trends observed in FW among monochromatic treatments in our study (Fig 4) were similar to those observed by [28] for relative quantum yield. The energy levels of red wavebands are optimal while those of green and blue are slightly higher than that required for photophosphorylation [28]. High-energy UV radiation can result in cell necrosis and damage to photosynthetic apparatus [2] leading to a decrease in quantum yield. On the other hand, far-red light is mainly absorbed by PSI [43] and insufficiently activates PSII leading to an imbalance between the two photosystems and an overall decrease in quantum yield [44].

Leaf number was lowest in the FR_733_ treatment (Fig 4 & Fig 5**, top**) similar to a shade avoidance syndrome [10]. Although plants received a light intensity of 200 µmol·m^-2^·s^-1^, a progressive increase in petiole and leaf elongation was observed in G_521_, R_632_, R_662_, and FR_733_ treatments, suggesting that plants experienced an increasing level of shade-like environment with decreasing waveband energy. There were no observed elongation responses in UV_389_ and B_451_, suggesting that a high energy level of photons can offset shade avoidance syndrome in plants.

### Short-term effects of UV exposure on phyllo levels and vegetative growth

The substitution of UV-A radiation during the stationary growth stage reduced lettuce FW by 24% (Table 2) but increased Phyllo level by 174% (Table 2). One of the morphological changes observed in plants in response to high-energy radiation is a decrease in leaf area, likely to minimize exposure to harmful radiation [10]. The exposure during the stationary growth stage likely decreased the size of newly formed leaves (which lowered biomass) and likely increased photoprotection by synthesizing more Phyllo. However, in older and fully grown leaves, which constitute the major portion of total LA, the likely protective mechanism is to synthesize Phyllo and other secondary metabolites that can aid in disseminating the excess energy and provide photoprotection to plants. This is likely the reason for a smaller reduction in biomass (with a non-significant reduction in dry matter) but a higher increase in Phyllo levels when plants are exposed to UV-A radiation for a short period during the stationary growth stage. An increase in carotenoids such as beta-carotene was observed when high-energy blue light was provided to plants during the stationary growth stages [37].

Revenue in indoor farming can increase when consumers pay higher prices for produce. Value-added traits such as increased levels of nutrients can attract consumers to buy more produce from indoor farms [4,5]. Therefore, a small reduction in vegetative growth but a large increase in value-added traits such as Phyllo levels may increase revenue in indoor farming. However, crop yields are a high priority in production and for this reason, stress-free environmental conditions are usually used to grow crops in indoor farming [45]. The inter-relations between vegetative growth and phytochemical biosynthesis are complex and enhancing one has shown to have negative consequences on the other [10,37]. In our study, we substituted 40% of total light with UV-A during the stationary growth stage. It may be possible to further lower biomass reduction while achieving a large increase in Phyllo levels by substituting a relatively smaller percentage of total light with UV-A (i.e., 20–25%). Optimizing the proportion of UV-A radiation during the stationary growth stage is important before including UV-A radiation in indoor farming. The current study did not investigate varietal-specific responses to UV-A substitution in broadband light on vegetative growth and Phyllo levels. Our goal for this research was to develop fundamental data on the short and long-term effects of UV-A radiation and associated physiological acclimation in lettuce. However, screening a range of lettuce varieties for tolerance to UV-A radiation may aid in identifying varieties with insignificant reductions in vegetative growth and significant increases in Phyllo levels.

### Role of increased phyllo: enhanced photoprotection vs photophosphorylation

Phylloquinone serves as an electron carrier in PSI [19] and an increase in its level could contribute to an increased electron transport through PSI and regeneration of NADPH needed for the downstream process of photosynthesis. This may suggest that an increase in Phyllo levels may enhance photophosphorylation. However, a significant reduction in the A_OP_ was observed in the UV_sub_ treatment (Table 3). Although not significant, the LCP was higher in UV_sub_ than in the control treatment. These are directly related to the observed lower LUE in UV_sub_ compared to the control. Analyses of A-*PPFD* curves revealed that A_OP_ at 200 µmol·m^-2^·s^-1^ of *PPFD* in the UV_sub_ treatment (2.4 µmol CO_2_·m^-2^·s^-1^) was similar to that observed at 120 µmol·m^-2^·s^-1^ of *PPFD* in the control (2.3 µmol CO_2_·m^-2^·s^-1^) based on the fitted equations (Fig 6, dashed lines). This may suggest that most of the UV-A light (i.e., 80 µmol·m^-2^·s^-1^) in the UV_sub_ treatment was not used in photochemistry and the observed A_OP_ in UV_sub_ mostly resulted from broadband growth lighting (i.e., 120 µmol·m^-2^·s^-1^) assuming minimal interactions between UV-A and growth lighting on photosynthesis. Based on this interpretation, increased Phyllo from UV-A exposure likely had little contribution to photophosphorylation.

**Fig 7:**
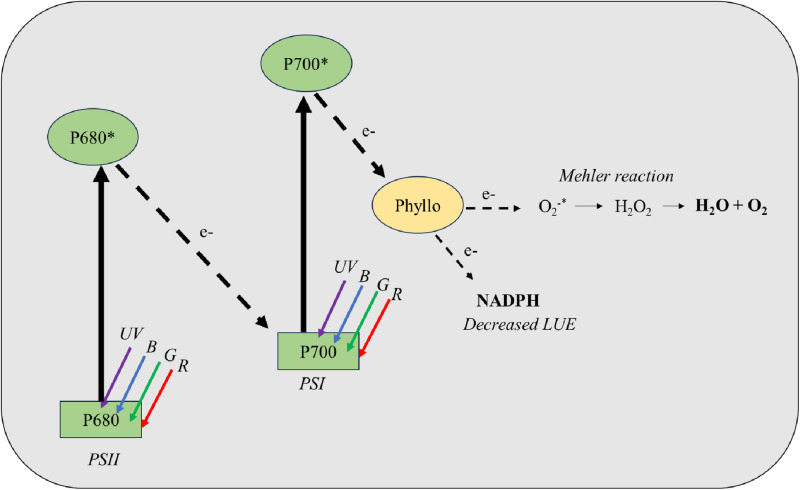
A simplified model of linear electron transport showing the role of phylloquinone in channeling electrons through the Mehler reaction when high-energy UV-A radiation is substituted to growth lighting. The increased flow of electrons from high-energy UV excitation through both photosystems results in an increased biosynthesis of phylloquinone which further increases the rate of Mehler reaction for photoprotection. The loss of electrons to the Mehler reaction reduces light use efficiency (LUE).

Exposure to UV radiation can result in the generation of reactive oxygen species (ROS) [46,47]. This can have harmful effects on PSII by damaging D1 and D2 proteins [48]. A decrease in dark-adapted quantum efficiency of PSII (F_v_/F_m_), an indicator of damage to photosynthetic apparatus, was reported in many studies when plants were exposed to UV-A radiation [2,26,27,49,50]. Under these conditions, plants generally synthesize compounds that can aid in scavenging ROS to protect them from excessive damage [51,52]. Energy (ATP) is needed for sub-cellular maintenance and repair of damaged organelles in plants [53,54], which is provided by respiration. Maintenance respiration can increase in response to damage to cellular organs [53] and results in higher respiration rates. The R_d_ was not statistically different between the control and UV_sub_ treatments (Table 3). This may suggest that damage due to high-energy UV radiation was minimal in the UV_sub_ treatment. Based on our results, we hypothesize that the short-term UV-A exposure can preferentially increase Phyllo for photoprotection in PSI (Fig 7). Increased Phyllo in PSI likely channeled excess electrons through alternate pathways such as the Mehler reaction. The loss of electrons, originally intended for NADPH generation, to alternate pathways reduces the rate of electron transport to photochemistry and lowers LUE (Table 3). Therefore, the observed increase in Phyllo in romaine lettuce cultivar Amadeus to short-term exposure to UV-A radiation during the stationary growth stage is likely a consequence of the tradeoff of photoprotection for photophosphorylation processes.

## Conclusions

Phylloquinone levels can be increased in lettuce by exposing plants to UV-A waveband (i.e., 380–390 nm) while other wavebands in the visible and far-red range have little effect on Phyllo. The enhancement of Phyllo from UV-A exposure is likely for photoprotection as opposed to increasing photophosphorylation. The long-term exposure to UV-A can have detrimental effects on lettuce plants. Short-term substitution of UV-A in broadband lighting during stationary growth can also increase Phyllo levels but lower lettuce vegetative growth. Although a relatively higher increase in the levels of Phyllo (174%) may potentially compensate for a smaller decrease in vegetative growth (24%) due to value addition, future research should screen for cultivar-specific effects to identify varieties with increased tolerance to UV-A radiation.
